# The Relationship between the Dominant Hand and the Occurrence of the Supracondylar Humerus Fracture in Pediatric Orthopedics

**DOI:** 10.3390/children8010051

**Published:** 2021-01-15

**Authors:** Alexandru Herdea, Alexandru Ulici, Alexandra Toma, Bogdan Voicu, Adham Charkaoui

**Affiliations:** 1 Pediatric Orthopedics Department, “Grigore Alexandrescu” Children’s Emergency Hospital, 011743 Bucharest, Romania; alexherdea@yahoo.com; 2 11th Department, “Carol Davila” University of Medicine and Pharmacy, 050474 Bucharest, Romania; 3 Department of Morphological and Functional Sciences, Faculty of Medicine and Pharmacy, “Dunărea de Jos” University of Galați, 800008 Galați, Romania; dr.alexandratoma@gmail.com (A.T.); charkaoui.adham@gmail.com (A.C.); 4 General Medicine Department, “Carol Davila” University of Medicine and Pharmacy, 050474 Bucharest, Romania; bogdanvoicu413@yahoo.com

**Keywords:** supracondylar humerus fracture, pediatric, humerus fracture, upper limb fracture, fracture laterality, handedness, pediatric orthopedics

## Abstract

It is known that during a fall, a child would rather protect their dominant hand by using the non-dominant one, although the role of handedness in upper limb fractures has not been studied in-depth. We carried out a retrospective, cross-sectional cohort study, including pediatric patients who presented to the emergency room with a supracondylar humerus fracture following an injury by falling from the same height. In total, 245 patients were selected and grouped according to age. In the 1–3 years group, no statistical significance was found between hand dominance and the side of fracture (*p* = 0.7315). During preschool years (4–6 years old), the non-dominant hand is more often involved (*p* = 0.03, odds ratio: 3.5). In the 7–14 years group this trend was maintained and actually increased (*p* = 0.052, odds ratio: 3.8). We might conclude that children tend to protect their dominant hand by falling on their non-dominant one. The main objective of this study is to highlight a link between handedness and the side of the body where the hand fracture will be identified in the pediatric population, regarding supracondylar humerus fracture.

## 1. Introduction

Supracondylar humerus fracture is a common type of elbow fracture in the pediatric population [1,2]. The peak incidence occurs between five and eight years, with a medium age of six years [3]. The literature findings reveal that two thirds of elbow fractures admitted to the hospital are supracondylar humerus fractures [1,4].

Supracondylar humerus fractures can result from traumatic events that interest the upper limb during both flexion and extension of the elbow, although hyperextension mechanism is more often incriminated [5]. They usually occur during sport and leisure activities as a result of falling from the same height or from under 3 m [6].

Complications of supracondylar humerus fracture include neurovascular injuries, compartment syndrome, delay of consolidation or myositis ossificans [7].

Handedness is known to be a factor involved in orthopedic pathologies besides the one being addressed by our study. In a record review of 169 patients with carpal tunnel syndrome, Reinstein found that it occurs significantly more often in the dominant hand than in the non-dominant one [8]. Goldberg et al. studied a group of 245 girls with idiopathic scoliosis with a minimum age of eight years and concluded that the lower thoracic curvature develops with the convexity towards the dominant side in 82% of the cases, with the correlation between scoliosis configuration and handedness being statistically significant [9]. Borton et al. found on a group of 426 children with unilateral fractures of the distal forearm that the overall risk of a fracture to occur on the non-dominant side was 57% [10]. The non-dominant side may be injury-prone simply because of the dominant hand that is involved in an ongoing activity or it is used to cling to an object in an attempt to prevent the fall [11].

Although there are many studies that address the management of such fractures and the efficacy of different treatments, only a few of them analyze the link between a child’s handedness and the side they are more likely to sustain a fracture [10,11,12].

Our study aims to highlight a link between the dominant hand and the side of a supracondylar humerus fracture in the pediatric population. The hypothesis is that children tend to guard their dominant hand in the event of a fall, thus exposing their non-dominant one to trauma because the dominant one might be involved in an activity.

## 2. Materials and Methods

The study took place in the Pediatric Orthopedics Department at “Grigore Alexandrescu” Children’s Emergency Hospital, Bucharest, Romania, located in an urban area, between May 2019 and November 2019. The ethics committee of “Grigore Alexandrescu” Children’s Emergency Clinical Hospital of Bucharest approved this study on 16 April 2019. The identification number of the study is 24/16 April 2019. An informed consent was obtained from the parents of all the participants. 

We performed a retrospective, cross-sectional cohort study that began on 30 May 2019 and ended on 12 November 2019.

Our inclusion criteria comprised: positive diagnosis (clinical examination along with elbow x-rays from both frontal and lateral views), trauma history (falling from the same height during recreational activities), age, sex and hand dominance. We excluded patients that had a history of falling from a different plane, high energy trauma, road accidents, incomplete patient data and lack of informed consent. Falling from the same height was defined as falling from a standing point as a result of slipping on a surface or tripping on objects no higher than knee-level, below 1.5 m.

The following data was acquired and analyzed: age, sex, side of fracture and dominant hand. The patients were classified by age in three groups: 1–3 years old (toddlers), 4–6 years old (preschoolers), 7–14 years old (schoolers). All of the patients included in the study group sustained a supracondylar humerus fracture with the elbow in extension. The Gartland classification and information about the therapeutic conduct were available but we considered it being irrelevant to the hypothesis of the study.

For the age group of 1–3 years old, handedness was established by asking the parents which hand does the child most often put in his mouth, and which hand is used to reach for objects whether they are given to him by someone else or not. In the 4–6 years old group, they were asked which hand is most often used to eat, play or draw. In the group of schoolers 7–14 years old, we asked which hand the child used for writing or brushing their hair.

Statistical analysis of data was performed through the GraphPad prism 6.1 and Medcalc 14 programs, where we calculated the odds ratio and statistical significance using Fisher’s exact test. A confidence interval of 95% was used and a *p* < 0.05 was considered statistically significant.

## 3. Results

A total of 403 patients presented to the emergency room in the selected time frame and were diagnosed with supracondylar humerus fracture. In total, 245 of the patients met the inclusion criteria. The remaining 158 patients were excluded due to incomplete data (49), lack of consent (35) or history of high energy trauma or road accidents (74).

Among the selected patients, 112 (45.7%) were girls, with a mean age of 5.25 years, and 133 (54.3%) were boys, with a mean age of 6 years.

The interrater reliability test that uses the dominant member as a decisional criterion and the laterality of fracture as the variability criterion demonstrates the lack of any direct, linear (*k* = −0.109) connection, thus refuting the test hypothesis of the occurrence of fractures in the dominant member. Practically, the test suggests the possibility that the fractures may appear in the non-dominant member, a hypothesis that we will test in the following research.

**Figure 1:**
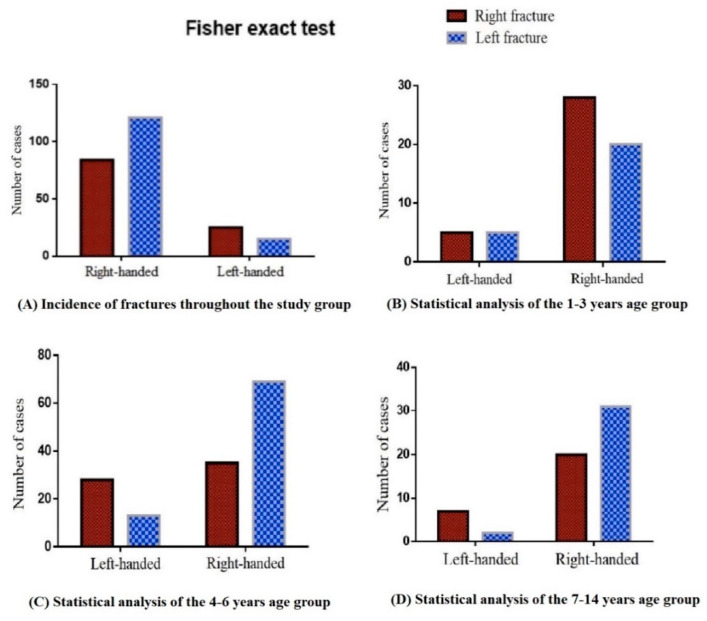
Results of Fischer’s exact test for the entire study group (**A**) and age group 1–3 (**B**), 4–6 (**C**) and 7–14 (**D**).

Following the main objective of this study, we performed a Fisher analysis that compared the frequency of fracture distribution by handedness. Thus, it can be seen in the graphical representation (Figure 1A) that patients tend to have non-dominant limb fractures (*p* = 0.01). Odds ratio analysis (OR = 2.41) can be interpreted as an independent risk factor for right-handed patients to undergo fractures on the left humerus, and for left-handed patients to undergo fractures on the right humerus.

There were 136 (55.5%) supracondylar fractures of the left humerus and 109 (44.5%) supracondylar fractures of the right humerus.

Fractures of the dominant hand occurred in 99 (40.4%) patients, while the rest of them sustained a fracture on the non-dominant side. Pertaining to right-handed patients, 121 (59%) of them sustained a fracture on the left side, while 84 (41%) of them had the right humerus fractured. Pertaining to left-handed patients, 15 (37.5%) of them sustained a fracture on the left side, while 25 (62.5%) of them had the right humerus fractured.

**Figure 2:**
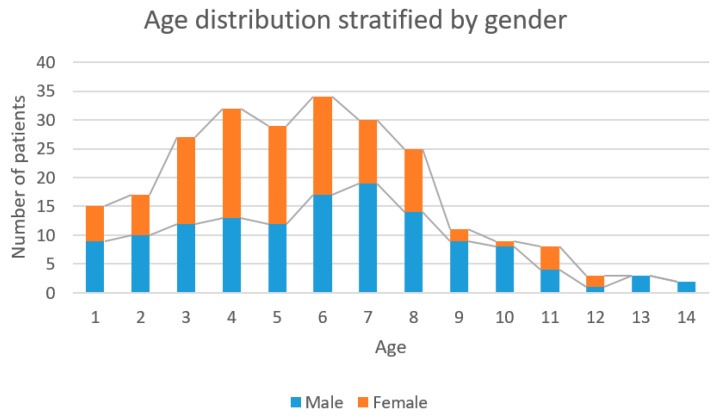
Age distribution stratified by gender.

The average age of the entire study group was 5.7 years and the median was 6 years, as seen in Figure 2. Using the D’Agostino and Pearson omnibus normality test, Wilcoxon Signed Rank Test and one sample *t* test it was found that the highest incidence of fractures was around the age of 6 (*p* < 0.0001).

In the age group of 1–3 years, there were 59 (24.1%) patients, 31 (52.5%) boys and 28 (47.5%) girls. In this group, 49 (83.1%) were right-handed children, 29 (59.2%) suffered a fracture on the right side and 20 (40.8%) on the left side. The remaining 10 (16.9%) patients were left-handed, five of them (50%) suffering a fracture on the right side and five of them (50%) on the left side. There was no statistical difference in fracture distribution among toddlers (*p* = 0.7315) pertaining to Fisher’s exact test (Figure 1B).

In the age group of 4–6 years, there were 95 (38.8%) patients, 42 (44.2%) boys and 53 (55.8%) girls. In this group, 77 (81.1%) were right-handed, 28 (36.4%) underwent supracondylar humerus fracture on the right side and 49 (63.6%) suffered a fracture on the left humerus. The remaining 18 (18.9%) were left-handed, 12 (66.7%) of them being diagnosed with supracondylar fracture of the right humerus and six (33.3%) of them having the left side involved (Figure 1C). Non-dominant upper limb fractures were best represented in this group with the correlation being statistically significant (*p* = 0.03) as a result of the Fisher’s exact test. The risk of non-dominant limb fracture, as calculated using the odds ratio, was 3.5 times higher than the risk of dominant hand fracture (OR = 3.5).

In the age group of 7–14 years, there were 91 (37.1%) patients, 60 (65.9%) boys and 31 (34.1%) girls, as shown in Figure 1D. There were 79 right-handed (86.8%) children, 27 (34.2%) of whom suffered a supracondylar fracture of the right humerus, the rest of the 52 patients (65.8%) having the left upper limb injured. There were 12 left-handed people (13.2%), 8 (66.7%) of whom sustained a right upper limb injury, the remaining four (33.3%) suffering a fracture of the left elbow. In this age group there is a clear tendency of fractures to occur in the non-dominant limb, but analyzing the frequency distribution using Fisher’s exact test, it was not statistically significant (*p* = 0.052). The risk of non-dominant limb fracture was 3.8 higher than the risk of dominant limb fracture (OR = 3.852) in schoolers.

In order to determine an age cut-off where non-dominant limb fractures begins to predominate, we performed a ROC analysis that uses the non-dominant limb fracture as a criterion for variability and age as a variable. Thus, the ROC analysis detected a three-year cut-off point, with a sensitivity of 82.9% and a specificity of 34.3% from which patients tend to sustain non-dominant supracondylar humerus fractures. Area under the ROC curve was close to 0.5 (AUC = 0.58), but due to the large number of patients in this study it was statistically significant (*p* = 0.03). Based on this analysis the establishment of the handedness below three years of age is irrelevant to the laterality of fracture.

## 4. Discussion

In order to correctly assess a supracondylar humerus fracture, clinical examination and conventional x-rays are needed [13]. Thus, the confusion between a supracondylar humerus fracture and a nursemaid’s elbow, another common elbow trauma in children under six, can be ruled out [14]. The treatment varies accordingly to the Gartland classification, ranging from closed reduction and casting to closed or open reduction and pinning using K wires [15]. In most severe cases, Computed Tomography followed by 3D reconstruction and 3D printing can help the orthopedic surgeon plan the safest surgery [16].

Vaquero-Picado et al. observed that this type of fracture occurs 1.5 times more often on the non-dominant limb than on the dominant one in male patients [3]. Calculating the same risk rate in our study population, we obtained a value of 2.41.

Given the relationship between the mean age of 5.7 years and the group’s median age of six years, we can surely say that the peak incidence of supracondylar humerus fractures occurs at six years old. This is consistent with Vaquero-Picado et al. who found the peak incidence of supracondylar humerus fractures to occur between five and eight years old, with the mean age being six years [3].

Studying data from the age group of 1–3 years shows no statistical significance between hand dominance and the laterality of fracture. We can say that in this case, a supracondylar humerus fracture may occur randomly and this is why we cannot continue to discuss possible risks at this age.

Data obtained from older age groups sustains the hypothesis that a supracondylar humerus fracture occurs more frequently on the non-dominant hand. Preschoolers are 3.5 times more likely to fracture their non-dominant hand. In this group, the correlation is statistically significant (*p* = 0.03). Preschoolers also tend to suffer non-dominant limb fractures, although the *p*-value was not statistically significant (*p* = 0.052). Mortensson et al. noticed that the dominant hand might be involved in an activity during a fall and that is why the traumatic event affects mainly the non-dominant hand [12].

One of the limitations we encountered in this study relates to the group of toddlers, as hand dominance could not be firmly established by the parents in some cases. Another drawback was the lack of ambidextrous children in this study as none presented to the pediatric orthopedic emergency room during our data collection. Fracture classification and preferred treatment were not taken into account.

Our objectives were met and the hypothesis that the non-dominant hand is more frequently injured in the event of elbow trauma was confirmed. The findings of our study were consistent with those of other researchers as Hassan [11] and Borton et al. [10].

## 5. Conclusions

The relationship between handedness and laterality of supracondylar humerus fractures is relevant only among children older than three years of age, because hand dominance starts to be relevant after this cut-off. Before the age of three, this type of fracture occurs as a random event as no statistical correlation was found.

Considering the above, we recommend the establishment of fracture prevention techniques during a fall accident so that the forces may be evenly distributed on both limbs whenever possible, avoiding the instinctive maneuver to fall on the non-dominant limb. These could be introduced in sport-related activities, both individual and collective.

Further studies can be conducted to evaluate this correlation according to the children’s activity preceding the traumatic event, as well as to monitor ambidextrous children.

## Data Availability

The data are not publicly available due to ethical reasons and patient privacy.
